# Design of Chitosan Sterilization Agents by a Structure Combination Strategy and Their Potential Application in Crop Protection

**DOI:** 10.3390/molecules26113250

**Published:** 2021-05-28

**Authors:** Weixiang Liu, Yukun Qin, Pengcheng Li

**Affiliations:** 1 CAS and Shandong Province Key Laboratory of Experimental Marine Biology, Center for Ocean Mega-Science, Institute of Oceanology, Chinese Academy of Sciences, Qingdao 266071, China; liuweixiang@qdio.ac.cn; 2 Laboratory for Marine Drugs and Bioproducts, Pilot National Laboratory for Marine Science and Technology (Qingdao), No. 1 Wenhai Road, Qingdao 266237, China

**Keywords:** chitosan derivative, structure combination strategy, modification, agriculture, antimicrobial application

## Abstract

Chitosan is the only cationic polysaccharide in nature. It is a type of renewable resource and is abundant. It has good biocompatibility, biodegradability and biological activity. The amino and hydroxyl groups in its molecules can be modified, which enables chitosan to contain a variety of functional groups, giving it a variety of properties. In recent years, researchers have used different strategies to synthesize a variety of chitosan derivatives with novel structure and unique activity. Structure combination is one of the main strategies. Therefore, we will evaluate the synthesis and agricultural antimicrobial applications of the active chitosan derivatives structure combinations, which have not been well-summarized. In addition, the advantages, challenges and developmental prospects of agricultural antimicrobial chitosan derivatives will be discussed.

## 1. Introduction

Chitosan, a natural basic amino polysaccharide prepared by deacetylation of chitin, is composed of β-(1→4)-linked d-glucosamine and *N*-acetyl-d-glucosamine units [1,2]. It mainly exists in the shells of marine animals such as shrimp and crabs and is a renewable marine biological resource with a large reserve [3,4]. As an environmentally friendly biomaterial, chitosan has a variety of biological activities, such as biocompatibility, biodegradation, biosafety, antimicrobial activities, coagulation activities and antitumor activities [5,6,7]. The United States Food and Drug Administration (FDA) has approved chitosan as safe for use in foods and drugs [4].

It has been reported that chitosan has broad-spectrum antimicrobial activity and can inhibit the growth of many kinds of bacteria, fungi, and yeast [8,9]. It can combine with the cell wall of microorganisms, inhibit the replication of DNA, and be used as a potential source of new biocides [10,11]. Therefore, in recent years, the application of chitosan in medicine, agriculture, food, textile and other fields has attracted much attention [12,13,14,15].

However, chitosan has a large number of stable hydrogen bonds in its molecular chain, which leads to its dissolution only in acidic solution and not at neutral pH, which is the main obstacle in its application. In addition, compared with chemical antimicrobials, chitosan has problems with a low efficacy and large dosage requirement [16]. Therefore, it is of great significance to improve the properties of chitosan by chemical modification. Due to the active amino and hydroxyl groups in chitosan molecules, alkyl [17], halogen [18], ester [19], sulfur [20], acyl [21], quaternary ammonium [22], carboxyl [23], phosphate [24], active aldehyde [25,26] and metal ions [27] can be introduced under certain conditions. Therefore, many bioactive molecules can be grafted onto chitosan to enhance the effects of chitosan and enhance the synergistic effects of chitosan and the grafted molecules [28]. In recent years, the preparation and properties of chitosan derivatives have become a new research direction [26,29].

In the past few years, there have been a large number of studies on chitosan derivatives. However, there are few reviews on the plant protection of chitosan derivatives based on an active structure combination strategy. Moreover, there are even fewer reviews on the structural splicing of chitosan derivatives and the applications of these derivatives in antimicrobial activities.

Therefore, this article will focus on the structural splicing methods and antimicrobial activities of chitosan derivatives and summarize the challenges and problems in the synthesis and application of these derivatives.

## 2. Structure Combination Strategy for Antimicrobial Chitosan Derivatives

Chitosan is rich in nature, and its C2-NH_2_, C3-OH and C6-OH in molecules can be chemically modified. However, due to steric hindrance, the reaction of C3-OH is difficult. Therefore, most of the grafting reactions of chitosan take place on C2-NH_2_ and C6-OH. After derivatization, the spatial structure of chitosan changes greatly; in particular, the crystal structure changes from ordered to amorphous, which leads to an increase in its solubility. The introduction of specific chemical groups into the active sites of chitosan can enhance the specific properties of the derivatives. Therefore, the chemical modification of chitosan is an effective method to improve the antimicrobial activity of chitosan. In recent years, the modification methods of chitosan have mainly included sulfonation, quaternization, phosphorylation, alkylation, Schiff alkalization, chelation and so on. After modification, the solubility and biological activity of chitosan are improved to a certain extent.

Many studies have shown that the introduction of antimicrobial groups into the chitosan skeleton could significantly improve its antimicrobial activity and broaden its antimicrobial spectrum. Due to the diversity of the grafted active molecule structures, their corresponding synthesis methods were also different. Therefore, the modification methods of chitosan will be discussed and summarized in this paper, which provides a theoretical basis and technical support for the development of new marine biological pesticides.

### 2.1. Introduction of Active Groups Containing Sulfur and Phosphorus

Organosulfur compounds and organophosphorus compounds are important synthetic intermediates that play an important role in medicine, pesticides and other fields. At present, they have been used as insecticides, herbicides and sterilizing agents, and increasing attention has been given to them. Therefore, the introduction of active groups containing sulfur and phosphorus on hydroxyl or amino groups may significantly improve the biological activity of chitosan derivatives.

Sulfonation is a common method for the synthesis of chitosan derivatives, which is economical and efficient technique (Table 1). Ewies et al. modified chitosan with benzoyl thiocyanate and ammonium thiocyanate to obtain a chitosan thiourea derivative. The derivative could effectively inhibit the germination of spores and sclerotia of *Beta vulgaris*, *Rhizoctonia solani* Kühn (AG_2-2_), *Sclerotium rolfsii* SACC and *Fusarium solani* (Mart.) SACC in the concentration range of 5 to 1000 μg/mL. The results showed that the chitosan thiourea derivatives had obvious inhibitory effects on the tested fungi [30,31].

In the study of Liu et al. [32], sulfonated chitosan (SCS) was prepared by nucleophilic substitution of 1,3-propane sultone with chitosan. The antifungal activities of the derivative in vitro and in vivo were determined by using *Botrytis cinerea* and *Arthrinium sacchari* as targets. The results showed that the inhibitory effect of SCS on *B. cinerea* was much higher than that on *A. sacchari*, which may have been due to the interaction between the discrete sulfonated chain of chitosan and because the *B. cinerea* cell membrane was more effective than those of chitosan and *A. sacchari*.

A large number of studies have shown that N-substituted thiourea chitosan derivatives have extensive antimicrobial activities. Mohamed et al. synthesized a variety of chitosan thiourea derivatives [33,34,35,36,37,38]. Among them, acyl thiorea carboxymethyl chitosan (CATUCMCS) had the best antifungal activity in vitro, and the MICs against *A. fumigate*, *G. candidum* and *A. albicans* were 7.8, 15.6 and 62.5 μg/mL, respectively.

With the bioisosterism approach, Zhong et al. and Qin et al. introduced sulfur-containing groups with high antifungal activity, such as sulfonamide, isothiocyanate, acyl thiosemicarbazide, thiosemicarbazide and dithiocarbamate, into chitosan molecules, and a variety of novel sulfur-containing chitosan derivatives were obtained [39,40,41,42,43,44]. Zhong et al. studied the inhibitory activities of chitosan sulfonamide derivatives, chitosan acyl isothiocyanate ester derivatives and chitosan acyl thiosemicarbazide derivatives on *Fusarium oxysporum* f. sp. *vasinfectum*, *Colletotrichum gloeosporioides* (*Penz.*) *Saec*, *Pseudoperonospora cubeneis* (*Berk et cort*) *Rostov* and other plant pathogenic fungi in vitro. The effects of the molecular weight and concentrations of the derivatives, steric effects of substituents, and types of substituents on their antifungal activities were discussed. It was found that the steric effects of substituents, molecular weight and electronegativity of derivatives had significant effects on the inhibition of plant pathogenic fungi. For example, the relative inhibition rate of the 200 kDa derivative (500 μg/mL) against *Alternaria solani* was 90%, while that of 57 kDa and 7 kDa was 46.67% and 40%, respectively. In addition, higher steric hindrance reduced the antifungal activity of the derivatives [40,44].

On this basis, Qin et al. introduced halogen and benzene groups into the sulfur-containing chitosan derivatives. It was found that electronegativity was still an important factor affecting the antifungal activity of the derivatives after halogen and benzene rings were introduced. The ionic absorption was weaker for chlorine than fluorine and trifluoromethyl, so its antifungal activity was stronger than that of the fluorine substituents and trifluoromethyl substituents, while trifluoromethyl had the strongest ionic absorption and the weakest antifungal activity. To obtain derivatives with stronger antifungal activity, Qin et al. introduced the active dithiocarbamate group of thiram into chitosan skeleton to prepare methyl dithiocarbamate chitosan and diethyl dithiocarbamate chitosan derivatives. The results showed that the dithiocarbamate group could improve the antifungal activity and broaden the antifungal spectrum of chitosan. At a concentration of 1000 μg/mL, the inhibitory activities of chitosan against *Gloeosporium theae sinensis* Miyake and *Alternaria porri* in vitro were 70.0% and 65.1% respectively, while the inhibitory rates of diethyl dithiocarbamate chitosan against the two pathogens were significantly increased, reaching 93.2% and 88.4%, respectively. Biological pesticides with good antifungal effects could be obtained by further improving the sulfur-containing derivatives of chitosan [42].

Organophosphorus compounds are widely used as agricultural sterilizing agents with obvious effects. Their basic chemical structures include phosphate esters, thiophosphates, phosphoamides and so on (Table 2). Among them, edifenphos [45], which can inhibit rice blast pathogens, and pyrazophos [46], a thiophosphate ester compound, have attracted extensive attention. Most of these organophosphorus sterilizing agents have the functions of internal absorption and have low levels of residues. However, long-term use of organophosphorus sterilizing agents will lead to resistance of pathogenic microbes. Therefore, it is necessary to modify organophosphorus compounds to reduce their side effects. Chitosan is a nontoxic natural polysaccharide and can have many active groups. By grafting phosphonate groups onto chitosan, the derivatives can both exhibit the fungicidal activity of organophosphorus and maintain the natural nontoxic side effects, biodegradability and good biocompatibility of chitosan.

**Figure 1:**
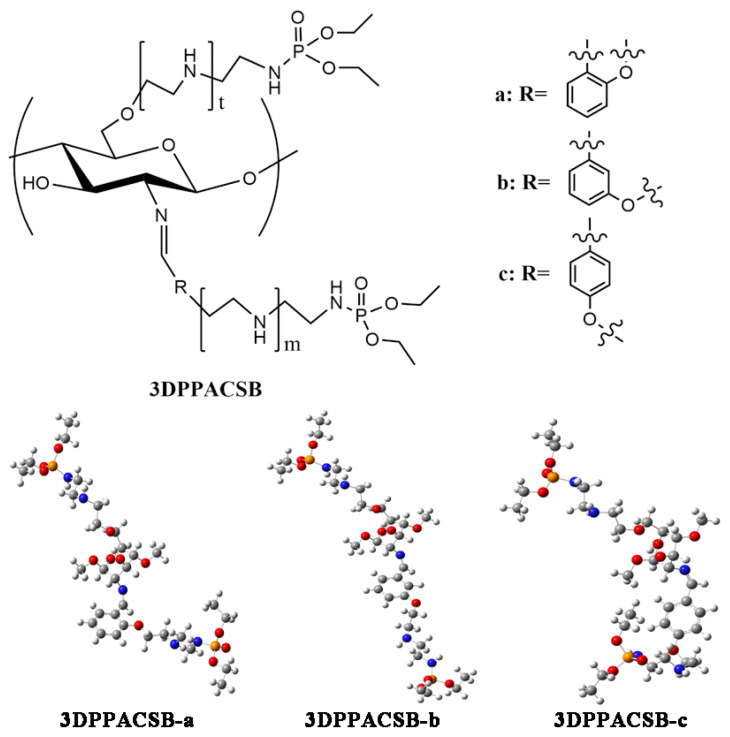
Molecular structure and unit geometry model of several phosphorylated chitosan derivatives (3DPPACSB: diethoxyphosphoryl polyaminoethyl chitosan Schiff bases).

Devarayan et al. synthesized N-(pdimethylaminobenzyl)-dimethyl-α-aminophosphonate chitosan and determined its antifungal activity against *Aspergillus niger* in vitro by a low-shear modeled microgravity [47]. The results showed that α-aminophosphonate chitosan had a significant inhibitory effect on the fungal growth under microgravity conditions. To improve the water solubility and antimicrobial activity of chitosan, Fan et al. grafted polyaminoethyl and diethoxyphosphoryl groups onto chitosan to obtain new water-soluble diethoxyphosphoryl polyaminoethyl chitosan derivatives [48] (Figure 1). The results showed that the in vitro antifungal activity of the derivatives increased with the increasing degree of substitution. This is because the phosphoryl groups in the molecule, as electron-withdrawing groups, can enhance the negative charge of polyamines, which made it easier for the protons to be absorbed and the cationic groups to form; thus, the antifungal activity of derivatives was enhanced.

Twenty kinds of chitosan derivatives with different molecular weights and different substituents were prepared by Zhong et al. [49]. The effect of alkyl chain length on the antimicrobial activity of chitosan derivatives containing phosphorus in vitro was studied. The antimicrobial activity of butyl phosphonate chitosan was found to be stronger than that of propyl phosphonate chitosan, and the activity of propyl substitution was stronger than that of ethyl substitution. The longer the alkyl chain was, the stronger the antimicrobial activity was. To determine the effects of substituents on the antimicrobial activity, Zhong et al. replaced the alkyl group with a benzene ring group containing different substituents at the α-site, and they discussed the relationship between the structure and antifungal activity of the derivatives. The results showed that the antifungal activity of the derivatives with α-substituted salicyl groups was higher than that of the derivatives with phenyl substituents due to the influence of the strong -OH electron donating group on the induction effect of the benzene ring, and their activity was better than that of the α-aminoalkyl phosphonate derivatives.

Qin et al. studied the effect of heterocyclic groups on the antifungal activity of chitosan derivatives containing phosphorus, and prepared (4-tolyloxy)-pyrimidyl-α-aminophosphates chitosan derivatives [50]. The results showed that these chitosan derivatives had a good antifungal effect in vitro and broad antifungal spectra. Compared with chitosan, the inhibition rate of α-aminophosphate chitosan derivatives on *Rhizoctonia solani Kühn* was 83.7% at 1000 mg/L, which was similar to that of polyoxin. The inhibitory effect of these derivatives on *Phomopsis asparagi* (*Sacc.*) and *Fusarium oxysporum* was especially effective. When the concentration was 250 mg/L, the inhibitory effect reached 100%, which was better than that of the positive control polyoxin. Therefore, these derivatives have the potential to be developed as new efficient, environmentally compatible, nonresidue biological pesticides.

### 2.2. Introduction of the Quaternary Ammonium Group

Due to solubility limitations, only the acidic chitosan solution had antimicrobial effects, while neutral and alkaline chitosan suspensions had no effects. In contrast, the quaternary ammonium salt of chitosan is soluble in water over a wide range of pH values, and its antimicrobial effect was better than that of chitosan. Quaternary ammonium salt of chitosan is one of the most important derivatives of chitosan. It is an ideal nontoxic antimicrobial material with high antimicrobial activity characteristics and a wide application range (Table 3).

**Figure 2:**
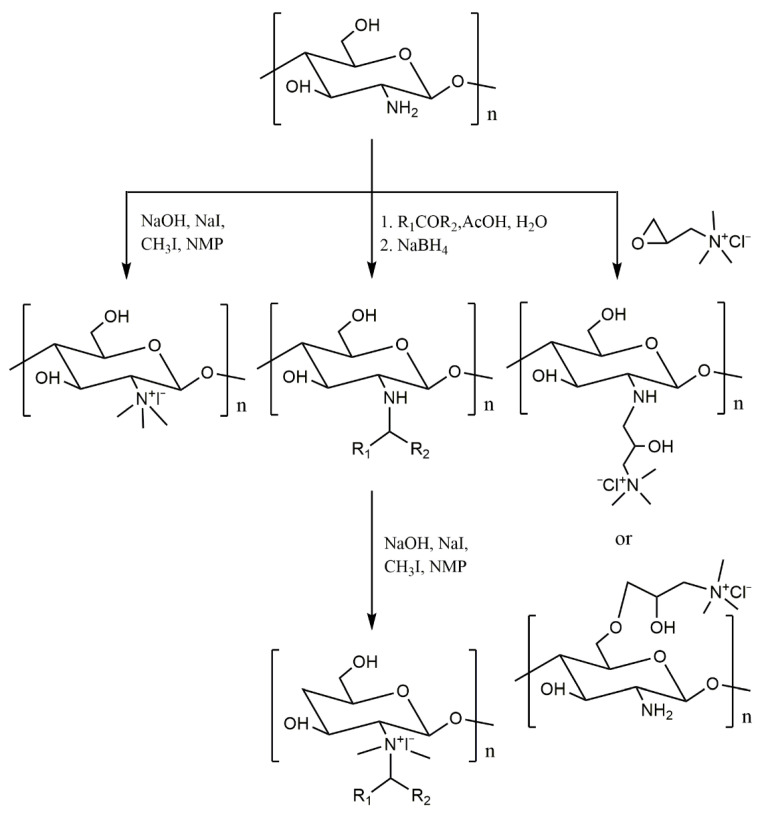
Three synthetic routes for quaternary ammonium salt of chitosan.

There are three main preparation methods for quaternized chitosan, as shown in Figure 2. In the first method, *N*,*N*,*N*-trimethyl chitosan quaternary ammonium salt was obtained by the rapid refluxing reaction of chitosan in *N*-methylpyrrolidone under the alkaline condition of sodium hydroxide and the catalysis of sodium iodide [51,52]. Although some O-methylated, N-methylated and *N*,*N*-dimethyl chitosan byproducts were produced, it was still the simplest and most efficient quaternization method. The second method is to form a Schiff base first and then perform a quaternization. The amino group of chitosan could react with aldehyde carbonyl or ketone carbonyl to form a Schiff base. The Schiff base was reduced by sodium borohydride to obtain *N*-alkyl chitosan derivatives. The quaternary ammonium salt of chitosan could be obtained by quaternization of *N*-alkyl chitosan derivatives with methyl iodide under alkaline conditions [53,54,55]. Using acetylacetone as the medium, Liu et al. grafted aminopyridine onto chitosan and then synthesized a variety of quaternary ammonium salts of chitosan. These derivatives had good growth inhibition effects on *Phytohthora capsici* and *Rhizoctonia solani* in vitro. TQCSP1 (chitosan derivatives with triple quaternary ammonium groups) inhibited the growth of *P. capsici* with inhibitory indexes of 91.94% at 0.8 mg/mL. In addition, it was also found that the positive charge of chitosan quaternary ammonium salt had a great influence on its antifungal activity. Generally, the stronger the positive charge is, the higher the antifungal activity [55]. The third method was to react chitosan with quaternary ammonium compounds. The active group of the compound could react with the amino or hydroxyl groups of chitosan to obtain the quaternary ammonium salt of chitosan. 2-hydroxypropyl-trimethyl ammonium chloride chitosan, which was prepared from the amino group or hydroxyl group of chitosan and 2,3-epoxypropyl trimethyl ammonium chloride, has been studied in more depth [56]. When reacted directly, *N*-substituted chitosan quaternary ammonium salt was obtained. When the amino group was protected first, O-substituted chitosan quaternary ammonium salt was obtained.

### 2.3. Introduction of Active Aldehydes or Ketones

**Figure 3:**
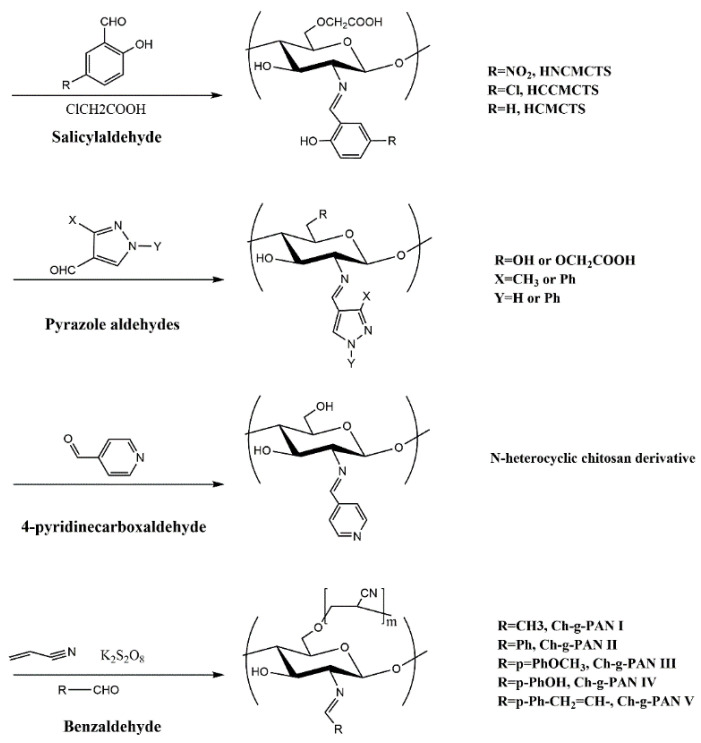
Schiff base derivatives of chitosan modified with active aldehydes or ketones.

Through a nucleophilic addition reaction, amino groups in chitosan molecules can react with an aldehyde carbonyl or keto carbonyl to obtain chitosan Schiff base derivatives, as shown in Figure 3. A variety of bioactive aldehydes or ketones can be grafted onto chitosan by Schiff base reaction. The obtained chitosan Schiff base derivatives have great application potential. On the one hand, Schiff base reaction can protect the amino group of chitosan so that the modification reaction occurs selectively on the hydroxyl group, making it is easy to remove the protective group and release the amino group under acidic conditions [57]. On the other hand, the amino groups of chitosan react with the aldehydes or ketones of high bioactivity groups and are reduced by sodium borohydride to obtain valuable N-substituted derivatives [58,59,60,61]. The N-substituted chitosan derivatives reduced by sodium borohydride can also be quaternized to obtain quaternary ammonium salt derivatives with good water solubility and antifungal or antioxidant activities [55].

The Schiff base reaction is a special reaction between an amino group and acyl group. Therefore, the reaction has good selectivity. It was reported that benzaldehyde [62,63,64], pyridinecarboxaldehyde [65,66], formylphenylboronic acid [67] and pyrazole aldehyde [68] can be successfully coupled with chitosan to obtain the corresponding Schiff base (Table 4). In addition, some chitosan Schiff base derivatives could also form hydrogels, which have both film-forming properties and antimicrobial activities.

### 2.4. Introduction of Metal Ions

Chitosan has been suggested to be the most powerful polymer with coordination abilities in natural products [27]. The amino groups in chitosan are mainly involved in coordination, and the nitrogen atom with a lone pair of electrons on the amino group is a very good electron donor. At the same time, the hydroxyl group in chitosan sometimes participates in coordination. The complexation mechanism of chitosan is very complex. The type of metal ions, pH of solution and main components of the solution can affect complexation. According to Lewis acid-base theory, when complexation occurs, metal ions (Lewis acids) act as electron acceptors, while chitosan molecules (Lewis bases) act as electron donors.

In recent years, the potential functions of chitosan metal complexes in agriculture, medicine and food have attracted extensive attention. It is well-known that chitosan and metal ions such as Ni^2+^ and Zn^2+^ have the functions of disinfection and sterilization [69,70]. When chitosan combines with metal ions through amino or hydroxyl groups, it may release some potential free electron donating atoms, which can enhance the antimicrobial activity [71]. Therefore, chitosan can gain enhanced antimicrobial activity after complexation with metal ions, which can promote its application in agriculture, medicine, food and other fields (Table 5).

The direct complexation of metal ions onto chitosan is an effective method for preparing chitosan metal complexes. At present, there are many studies on this kind of metal complexes. Khan et al. synthesized chitosan zinc complexes under gamma irradiation [72]. These complexes showed excellent antifungal activity in vitro and inhibited the growth of *Fusarium solani*, even after two weeks.

**Figure 4:**
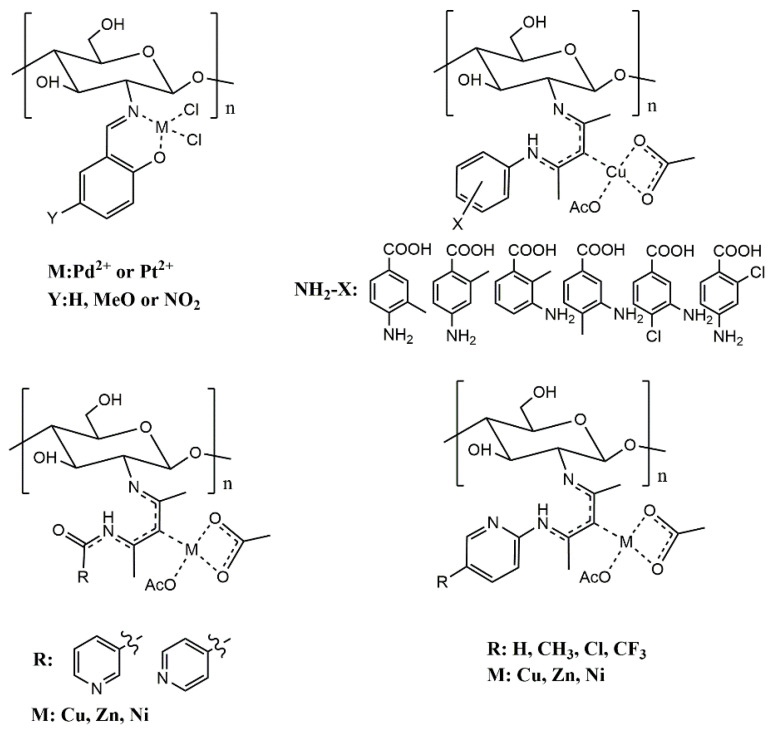
Chitosan metal complexes with antimicrobial activity.

In addition to direct complexation, chitosan metal complexes can also be prepared by chemically modifying chitosan to improve its properties then complexing the metal ions. The composites prepared by this method often have different characteristics due to the different modification methods. At present, the preparation of chitosan metal complexes by modification complexation method is still in the development stage, and there are few studies in this field (Figure 4). Goncalves et al. prepared a chitosan salicylaldehyde palladium complex and chitosan salicylaldehyde platinum complex using chitosan and three kinds of salicylaldehydes. Compared with chitosan, the chitosan complex displayed high toxicity to the MCF-7 cell line and inhibitory effects on *Pseudomonas syringae pv. Tomato* in vitro [25]. The author of this paper used acetylacetone to connect chitosan with aminobenzoic acid, nicotinamide and aminopyridine. Then, O-carboxymethyl chitosan Schiff base derivatives were obtained by O-carboxymethylation, and they were chelated with metal ions to obtain O-carboxymethyl chitosan Schiff base metal complexes. These complexes had good water solubility and showed strong antifungal activity in vitro [16,73,74]. In the pot experiment, the disease indexes of pepper seedlings irrigated with chitosan derivatives were significantly lower than those of the negative control group irrigated with water. At a concentration of 0.8 mg/mL, O-CSP4-Cu (*O*-carboxymethyl chitosan copper complexes) showed the strongest antifungal activity in vivo, and the protective efficacy and curative efficacy were 85.56% and 74.41%, respectively, which were slightly higher than that of the positive control cuproxat [74].

The modification of chitosan by nanotechnology is also an important method to prepare antifungal chitosan metal derivatives [75]. This method can effectively avoid the influence of organic solvents on the antifungal activity of the derivatives [29]. Rubina et al. prepared chitosan-copper nanocomposites by a metal vapor synthesis (MVS) technique. High concentrations of Cu@Chit NC (copper-carrying chitosan nanocomposites) that were prepared using acetone as a solvent could significantly inhibit the growth of *Sclerotium rolfsii* and *Rhizoctonia solani* AG-4 in vitro. In addition, some phenomena, such as the loss of the cytoplasm content, cytoplasmic coagulation, irregular shape of mycelia, or destruction in the hyphae, were observed [76]. Other researchers have also synthesized a variety of chitosan copper nanocomposites (chitosan-coated copper nanoparticles, copper-silica-chitosan nanoparticles, Cu-chitosan, Cu-chitosan nanoparticles) by different methods and found that they had good inhibitory effects on plant pathogenic fungi such as Pythium aphanidermatum, Botrytis cinerea, Rhizoctonia solani and *Curvularia lunata* in vitro and in vivo [77,78,79,80]. These nanomaterials based on chitosan can promote the development of sustainable agriculture and have good commercial development potential in the field of plant protection.

### 2.5. Introduction of Alkyl

In addition to the above strategies, alkylation is also an effective method for chitosan modification, which mainly occurs on the amino and hydroxyl groups of chitosan (Table 6). Three kinds of alkylated chitosan derivatives—*O*-alkyl chitosan, *N*-alkyl chitosan and *N*,*O*-alkyl chitosan—can be formed. The solubility of chitosan derivatives is related to the length of the alkyl chain.

**Figure 5:**
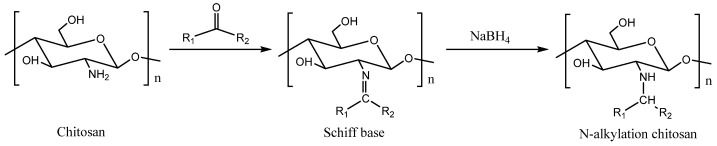
Preparation of N-alkylated chitosan by the Schiff base method.

For *O*-alkylated chitosan, the Schiff base method (Figure 5), N-phthaloylation method and metal template synthesis method are usually used [81]. For example, Su et al. grafted arginine onto the C_6_ site of chitosan by a Schiff base reaction between benzaldehyde and chitosan to protect the amino group. 6-Deoxy-6-arginine-modified chitosan (DAC) was thus prepared [57].

The C_2_ amino group in the chitosan molecule belongs to the first-order amino group, which has a strong nucleophilic property, and the activity of the amino group is higher than that of the hydroxyl group, so it is easier to introduce substituents into the chitosan molecule by *N*-alkylation. *N*-alkylation derivatives can be prepared by the Schiff base method. Aldehydes or ketones can react with free amino groups of chitosan by Schiff base reaction, and the double bond in molecules can be reduced by NaBH_4_ or KBH_4_. The corresponding *N*-alkylated chitosan can then be obtained [58,59,60,61]. For example, Rabea et al. grafted a variety of aromatic aldehydes and aliphatic aldehydes onto the amino groups of chitosan and prepared more than 20 kinds of *N*-alkylated chitosan with different chain lengths. The alkylated chitosan derivatives had good growth inhibition effects on many plant pathogenic fungi. The EC_50_ of *N*-(2,6-dichlorobenzoyl)chitosan on *Botrytis cinerea* Pers was 0.52 g/L, and the inhibition rate of *N*-(*m*-nitrobenzoyl)chitosan on *Pyricularia grisea* Cavara was 77% at a concentration of 5 g/L. In addition, some of the derivatives had good insecticidal activity against the cotton leafworm *Spodoptera litoralis* [58,59]. Jagadish et al. and Badawy et al. synthesized *N*-vanillyl chitosan and *N*-(cinnamyl) chitosan analogs by the Schiff base method. The results showed that the tensile strength, water vapor permeability and haze properties of *N*-vanillyl chitosan were significantly changed. The aflatoxin produced by *Aspergillus flavus* was reduced by 98.9% with modified chitosan [60]. The EC_50_ values of N-(o-methoxycinnamyl) chitosan against *Alternaria alternata*, *Botrytis cinerea*, *Botryodiplodia theobromae* and *Phytohthora infestans* were 672, 796, 980 and 636 mg/L, respectively. Thus, it is a potential safe substitute for chemical fungicides [61].

*N*,*O*-substituted chitosan derivatives can be prepared by the direct reaction of chitosan with haloalkanes under alkaline conditions [82]. The solubility of derivatives is affected by changes in the reaction conditions. These derivatives have good biocompatibility and are often used in medical materials.

## 3. Conclusions and Outlook

Chitosan, a renewable macromolecular polysaccharide with extensive sources and a large supply, mainly comes from abandoned shrimp and crab shells, and it is a typical biological resource for waste reuse. Chitosan not only has good biocompatibility, biodegradability and natural nontoxic characteristics, but also has unique amino structure and polycation properties. Therefore, chitosan is considered to be the only alkaline polysaccharide and cationic polysaccharide in nature. Based on the unique source, structure and properties of chitosan, and as an active material or biomaterial, chitosan has great potential for development and application in agriculture, food, medicine, cosmetics and other fields.

However, due to its high molecular weight, poor solubility and weak bioactivity, chitosan is difficult to develop into biomaterials and products that can be directly applied. Therefore, the structure of chitosan can be modified by chemical methods to induce synergistic interactions between the grafted active groups and chitosan. A variety of chitosan derivatives can be prepared by sulfonation, quaternization, Schiff basification, and metal complexation reactions. Different active groups can be grafted onto chitosan molecules, and chitosan derivatives with high antimicrobial activity can be obtained through activity screening, which is the main research direction in the application of chitosan derivatives.

In recent years, with the rapid development of global agriculture, people’s demand for green and healthy food has also increased, which brings great challenges to the chemical control of plant diseases. The residues of traditional organic chemical pesticides usually bring risks of toxicity, carcinogenesis and teratogenicity. The demand for the replacement of agricultural pesticides is becoming increasingly urgent. Research on new biological pesticides has become an urgent task for the prevention and control of plant diseases. Chitosan derivatives are promising plant protection and plant growth promoters. They can not only effectively improve the defense ability of plants against pathogens but also inhibit the growth of pathogenic microorganisms. This kind of pesticide based on biological materials can provide effective support for the sustainable development of agriculture and has good application prospects.

## Figures and Tables

**Table 1:** Antifungal properties of some sulfonated chitosan derivatives.

Sample	Molecular Weight	Sulfur Content	Grafting Degree	Antifungal Activity ^a^	References
CATUCS	50 kDa	9.83%	91.5%	*Colletotrichum gloeosporioides* (*Penz.*) *Saec* (67%) at 500 μg/mL in vitro	[39]
A(*p*-T)TSCZCS	200 kDa	7.44%	35.3%	*Aiternaria solani* (93%) at 500 μg/mL in vitro	[40]
CATUCMCS	200 kDa	7.56%	88.88%	*Aspergillus fumigate* (MIC: 7.8 μg/mL) in vitro	[33]
o-HPHTCNCS	230 kDa	0.81%	8.62%	*Rhizoctonia solani Kühn* (100%) at 500 μg/mL in vitro	[41]
CA(*o*-MP)TSCZCS	200 kDa	6.73%	33.7%	*Alternaria Solani* (82.35%) at 500 μg/mL in vitro	[43]
PIBTU-CS-4	200 kDa	7.00%	88%	*Geotricum candidum* (MIC: 3.9 μg/mL) in vitro	[38]

^a^ % indicates the antifungal index (%), while minimum inhibitory concentration (MIC) is expressed as μg/mL. CATUCS: chloracetyl derivative of chitosan; A(p-T)TSCZCS: acetyl phenyl–thiosemicarbazone–chitosan; CATUCMCS: acyl thiourea carboxymethyl chitosan derivative; o-HPHTCNCS: o-hydroxyphenylaldehyde thiosemicarbazone chitosan; CA(o-MP)TSCZCS: chloracetyl phenyl-thiosemicarbazone-chitosan; PIBTU-CS-4: *N*,*N*’-bis[4-(chlorocarbonyl)phenyl] pyromellitimide chitosan.

**Table 2:** Antifungal properties of some phosphorylated chitosan derivatives.

Sample	Method	Target Fungi	Antifungal Activity ^a^	References
*N*-(p-dimethylaminobenzyl)-dimethyl-α-aminophosphonate chitosan	Radial hyphal growth bioassay in vitro	*Aspergillus niger*	76% at 500 μg/mL	[47]
Diethoxyphosphoryl polyaminoethyl chitosan Schiff bases	Plate growth rate method in vitro	*Phytophthora capsici* Leonian	89% at 0.8 mg/mL	[48]
2-(α-arylamino phosphonate)-chitosan	Plate growth rate method in vitro	*Fusarium oxysporum* f. sp. *vasinfectum*	95.24% at 500 μg/mL	[49]
4-(pyrimidyl-2-yloxy)benzaldehyde and (4-tolyloxy)-pyrimidyl-α-aminophosphonates chitosan	Mycelium growth rate test in vitro	*Fusarium oxysporum*, *Phomopsis asparagi* (*Sacc.*)	100% at 250 μg/mL	[50]

^a^ % indicates the antifungal index (%).

**Table 3:** Antifungal properties of some chitosan quaternary ammonium salts.

Sample	Method	Target Fungi	Antifungal Activity ^a^	References
Chitosan derivatives with triple quaternary ammonium groups	Mycelium growth rate test in vitro	*Phytophthora capsici*, *Rhizoctonia solani*	91.94% against *P. capsici* and 87.18% against *R. solani* at 0.8 mg/mL	[55]
*N*,*N*,*N*-(diethyl-p-dimethylaminobenzyl) chitosan	Detached leaf method in vivo	*Botrytis cinerea*	100% at 1 mg/mL	[54]
Quaternized 6-oxychitosan derivatives	Plate growth rate method in vitro	*Verticillium albo-atrum*	89.1% at 0.4 μg/mL	[53]
*N*,*N*,*N*-trimethyl-*O*-(ureidopyridinium)acetyl chitosan derivatives	Mycelium growth rate test in vitro	*Phomopsis asparagus*	80.65% at 1 mg/mL	[52]

^a^ % indicates the antifungal index (%).

**Table 4:** Antifungal properties of some chitosan quaternary ammonium salts.

Sample	Method	Target Fungi	Antifungal Activity ^a^	References
2-(2-hydroxybenzylideneamino)-6-carboxymethyl-chitosan	Mycelium growth rate test in vitro	*Valsa mali*	83% at 500 μg/mL	[62]
Chitosan-graft-poly(acrylonitrile) Schiff bases	Filter paper diffusion method in vitro	*Geotricum candidum*	MIC: 1.95 μg/mL	[64]
O-carboxymethyl chitosan Schiff base	Plate growth rate method in vitro	*Phytophthora capsici*	49.8% at 0.2 mg/mL	[16]

^a^ % indicates the antifungal index (%), while minimum inhibitory concentration (MIC) is expressed.

**Table 5:** Antifungal properties of some chitosan metal complexes and nanocomposites.

Sample	Method	Target Fungi	Antifungal Activity ^a^	References
Chitosan-zinc complexes	Mycelium growth rate test in vitro	*Aspergallious fumigatus*, *Fusarium solani*	No growth after two weeks	[72]
Chitosan ortho-hydroxyaryl Schiff base palladium complex	Colorimetric method in vitro	*Pseudomonas syringae* pv. *tomato*	MIC: 25 μg/mL IC_50_: 7 μg/mL	[25]
*O*-carboxymethyl aminobenzoic acid chitosan Schiff base Cu complex	Plate growth rate method in vitro	*Phytophthora capsici*	100% at 0.2 mg/mL	[16]
***O***-carboxymethyl nicotinamide chitosan Schiff base metal complexes	Plate growth rate method in vitro	*Phytophthora capsici*, *Botrytis cinerea*	100% at 0.2 mg/mL	[73]
***O***-carboxymethyl aminopyridine chitosan Schiff base metal complexes	Pot experiment in vivo	*Phytophthora capsici*	85.56% for protective efficacy and 74.41% for curative efficacy at 0.8 mg/mL	[74]
Copper-carrying chitosan nanocomposites	Agar medium assay in vitro	*Sclerotium rolfsii*	100% at 0.1 mg/mL	[76]
Chitosan-coated copper nanoparticles	Mycelium growth rate test in vitro	*Rhizoctonia solani*, *Pythium aphanidermatum*	94.3% against *R. solani* and 98.3% against *P. aphanidermatum* at 0.1%	[77]
Copper-silica-chitosan nanoparticles	Plate growth rate method in vitro	*Botrytis cinerea*	43% at 4 mg/mL	[78]
Cu-chitosan nanocomposites	Mycelium growth rate test in vitro	*Curvularia lunata*	52.7% at 0.16%	[80]

^a^ % indicates the antifungal index (%), while half inhibitory concentration (IC_50_) is expressed.

**Table 6:** Antifungal properties of some alkylated chitosan derivatives.

Sample	Method	Target Fungi	Antifungal Activity ^a^	References
*N*-(2,6-dichlorobenzyl)chitosan	Plate growth rate method in vitro	*Botrytis cinerea* Pers	EC_50_: 0.52 g/L	[58]
*N*-(2,2-diphenylethyl)chitosan	Plate growth rate method in vitro	*Botrytis cinerea* Pers	EC_50_: 0.031 g/L	[59]
*N*-vanillyl chitosan	Direct exposure method in vitro	*Aspergillus flavus*	98.9%	[60]
*N*-(cinnamyl) chitosan analogs	Mycelial radial growth technique in vitro	*Phytophthora infestans*	EC_50_: 636 mg/L	[61]

^a^ % indicates the antifungal index (%), while concentration for 50% of maximal effect (EC_50_) is expressed.

## Data Availability

Not Applicable.
